# A Web-Based Game for Young Adolescents to Improve Parental Communication and Prevent Unintended Pregnancy and Sexually Transmitted Infections (The Secret of Seven Stones): Development and Feasibility Study

**DOI:** 10.2196/23088

**Published:** 2021-01-27

**Authors:** Ross Shegog, Laura Armistead, Christine Markham, Sara Dube, Hsing-Yi Song, Pooja Chaudhary, Angela Spencer, Melissa Peskin, Diane Santa Maria, J Michael Wilkerson, Robert Addy, Susan Tortolero Emery, Jeffery McLaughlin

**Affiliations:** 1 Department of Health Promotion and Behavioral Sciences School of Public Health University of Texas Health Science Center Houston Houston, TX United States; 2 Mathematica Policy Research Princeton, NJ United States; 3 The Widen Lab University of Texas at Austin Austin, TX United States; 4 School of Biomedical Informatics University of Texas Health Science Center at Houston Houston, TX United States; 5 Special Supplemental Nutrition Program for Women, Infants and Children (WIC) Washington, DC United States; 6 School of Nursing University of Texas Health Science Center Houston Houston, TX United States; 7 Radiant Digital, LLC Vienna, VA United States

**Keywords:** serious game, intervention mapping, sexual health, adolescents, sexually transmitted infections, teenage pregnancy, parent, communication, intergenerational, mobile phone

## Abstract

**Background:**

Early adolescent unintended pregnancy and sexually transmitted infection prevention are significant public health challenges in the United States. Parental influence can help adolescents make responsible and informed sexual health decisions toward delayed sexual debut; yet parents often feel ill equipped to communicate about sex-related topics. Intergenerational games offer a potential strategy to provide life skills training to young adolescents (aged 11-14 years) while engaging them and their parents in communication about sexual health.

**Objective:**

This study aims to describe the development of a web-based online sexual health intergenerational adventure game, the *Secret of Seven Stones* (SSS), using an intervention mapping (IM) approach for developing theory- and evidence-based interventions.

**Methods:**

We followed the IM development steps to describe a theoretical and empirical model for young adolescent sexual health behavior, define target behaviors and change objectives, identify theory-based methods and practical applications to inform design and function, develop and test a prototype of 2 game levels to assess feasibility before developing the complete 18-level game, draft an implementation plan that includes a commercial dissemination strategy, and draft an evaluation plan including a study design for a randomized controlled trial efficacy trial of SSS.

**Results:**

SSS comprised an adventure game for young adolescent skills training delivered via a desktop computer, a text-based notification system to provide progress updates for parents and cues to initiate dialogue with their 11- to 14-year-old child, and a website for parent skills training and progress monitoring. Formative prototype testing demonstrated feasibility for in-home use and positive usability ratings.

**Conclusions:**

The SSS intergenerational game provides a unique addition to the limited cadre of home-based programs that facilitate parent involvement in influencing young adolescent behaviors and reducing adolescent sexual risk taking. The IM framework provided a logical and thorough approach to development and testing, attentive to the need for theoretical and empirical foundations in serious games for health.

## Introduction

### Background

Early sexual debut in adolescents is a pervasive public health challenge in the United States. Nearly half (46.8%) of high school students reported having engaged in sexual intercourse, whereas 5.6% reported having engaged in sex before the age of 13 years [1]. Furthermore, teenagers and young adults (aged 10-24 years) represent approximately 90% of single parents and half of all new cases of sexually transmitted infections (STIs) [2,3]. Sexuality and gender identity typically emerge during early adolescence around 10 to 14 years of age, a period corresponding with early experimentation with sexual behaviors [4]. However, it is also a time of receptivity to health messages and therefore opportune for interventions to positively influence future sexual health decision making [4].

School- and clinic-based prevention programs often achieve broad support and success in reducing sexual risk behaviors in young adolescents [5-14]. Conversely, these programs often face implementation barriers such as perceptions of sex education as controversial, limited time and resources, and lack of fidelity [14-17]. Furthermore, school- and clinic-based programs face challenges in effectively involving parents as an unintended pregnancy and STI and HIV prevention mediator. A growing body of research supports the importance of parents’ influence on young adolescent risk behaviors; however, many parents express reluctance to discuss sex with young adolescents because of the belief that they lack time, knowledge, or appropriate skills [18-20]. Young adolescents also report a desire to discuss sexual health–related topics with their parents but feel that their parents need training on how to communicate about these topics [21]. Web-based and mobile technology may provide utility in reaching young adolescents and parents with novel sexual health skills training programs [22-24]. Serious gaming offers promise as an innovative and efficacious approach to sexual health education, operating as a forum to promote a common intergenerational experience, and as a catalyst for increased communication [25-36]. Recent research on parent-based adolescent sexual health education suggests promise for the use of intergenerational games in enabling collaborative engagement across age groups [37].

### Purpose

The purpose of this study is to describe the development of a novel in-home web-based intergenerational game for parent and young adolescent (11-14 years) dyads, the *Secret of the Seven Stones (SSS),* designed to provide sexual health skills training to young adolescents and to positively impact dyadic sexual health communication. SSS is played on internet-accessible devices through the Adobe Air framework and comprises 18 game levels (each of 45 min of game play) with 50 interactive skills training clusters, 54 card *battle* sequences, and 7 game-mediated parent-young adolescent partner-engage-plan (PEP) talks. Players adopt an avatar to negotiate the town of Seven Stones and assume a hero’s quest to liberate the inhabitants when their personal life rules are challenged in contexts of maintaining healthy friendships, understanding puberty and reproduction, having healthy dating relationships, refusing sex, and negotiating safe sexual practices. The player must *power-up* their wisdom, skill, and support capabilities in these domains (represented in the game as battle cards) in a dojo using skill training and rehearsal mini-games, animations, puzzles, quizzes, role modeling, and peer video. The player can then liberate an inhabitant by winning a card *battle*, releasing them from misperceptions, poor judgment, and bad decision making. At 7 milestones in the game, the player is cued to have a PEP talk with their collaborating parent who is a gatekeeper, conferring a code that enables continuation of the quest. PEP talks focus on the concepts and strategies covered in the game and also introduces each of the 7 character traits that are important in navigating one’s life decisions (eg, respect, vision, persistence, caring, responsibility, courage, and integrity). Throughout the game, SMS text prompts notify the parent of the player’s progress, cue them to an upcoming PEP talk, and link them to parent website resources that can assist with the PEP talk, providing progress tracking, supporting videos, and downloadable fact sheets. Theoretical methods and practical applications guide behavioral skills training that draws from 135 performance behaviors and over 1300 learning objectives within 15 sexual health domains encompassing responsible decision making about communication, friendships, dating relationships, sex, and social support. SSS was developed using the intervention mapping (IM) framework [38]. The development process is described in the context of each of the six IM steps.

## Methods

### Overview

We developed SSS through a National Institutes of Health (NIH) Small Business Technology Transfer Research (STTR) project collaborative between UTHealth (The University of Texas Health Science Center Houston) and Radiant Creative Group, LLC. Our development team comprised specialists in adolescent sexual health, computer-based interventions, parent-child communication, and digital media development. The Parent-Youth Advisory Group (P-YAG) provided conceptual guidance and formative evaluation. Parents (n=20) and young adolescents (n=19, aged 11-14 years) were recruited through flyers, targeted Facebook advertisements, and word of mouth. Young adolescents were mainly female (13/19, 68%), mean 12 (SD 0.28) years old, African American (9/19, 47%), and White (8/19, 42%). Parents were mainly mothers (17/20, 85%), African American (8/20, 40%), White (9/20, 45%), and Hispanic (3/20, 15%). IM, a 6-step framework for developing evidence- and theory-based intervention programs, guided our development process (Table 1) [38]. Our study was approved by the University of Texas UTHealth Institutional Review Board. At the initial P-YAG meeting, parents and young adolescents signed consent and assent forms, respectively.

**Table 1:** Intervention mapping steps with associated tasks and intermediate development products.

IM^a^ steps	IM tasks	Intermediate development products^b^
Step 1: Assess need & develop a logic model of the problem	Establish and work with a planning group. Describe the context for the intervention, including the population, setting, and community. Conduct a needs assessment to create a logic model of the problem.	P-YAG^c^ Literature review–evidence table PRECEDE^d^ model
Step 2: Develop matrices of change objectives	State expected outcomes for behavior and environment. Specify performance objectives for behavioral and environmental outcomes. Select determinants for behavioral and environmental outcomes. Construct matrices of change objectives.	Matrices for parent (n=6), youth (n=8), and dyadic (n=1) outcome behaviors comprising performance objectives for parent (n=65), youth (n=70), and dyad (n=8) and learning objectives for parent (n=869), youth (n=781), and dyad (n=72). Conceptual model for SSS^e^ game flow (model of change).
Step 3: Identify theory-based methods and practical applications for program design	Choose theory- and evidence-based methods to create change. Select or design practical applications to deliver change methods. Generate program themes, channels, components, scope, and sequence.	Table of content domains (n=9). SSS design document comprising specifications including functional inventory, game flow, screen map design, game mechanics, scripts, character descriptions, and interactive activities.
Step 4: Produce program components and materials	Refine program structure and organization. Prepare plans for program materials. Draft messages, materials, and protocols. Pretest, refine, and produce materials.	SSS game consisting of 18 levels of content. SSS parent website including parent training videos (n=7) and tip sheets (n=10). Pilot test protocols and results: Manual of procedures. Usability rating results table (parent & youth) with ratings on ease of use, acceptability, credibility, motivational appeal, and applicability for 2 prototype levels. Qualitative data (parent and youth) on acceptability for in-home use.
Step 5: Plan for program adoption, implementation, and sustainability	Identify potential program implementers. State outcomes and performance objectives for implementation. Construct matrices of change objectives for implementation. Design implementation interventions.	Marketing and commercialization plan for future implementers. Written University of Texas Tech Transfer agreement. SSS game and website revisions for future implementation.
Step 6: Plan for evaluation	Write effect and process evaluation questions. Develop indicators and measures for assessment. Specify evaluation design.	Efficacy study design Manual of Procedures comprising: Study hypotheses and protocols. Baseline and first and second follow-up Questionnaire Development System (QDS) software and paper-based surveys. Qualitative exit interview prompts.

^a^IM: intervention mapping.

^b^Youth indicates young adolescents (11-14 years).

^c^P-YAG: Parent-Youth Advisory Group.

^d^PRECEDE: predisposing, reinforcing, and enabling constructs in educational diagnosis and evaluation.

^e^SSS: Secret of Seven Stones.

### Intervention Development

IM is a stepped framework to guide the development of behavioral interventions, providing a process by which program developers can apply social and behavioral science theories within the practice of health behavior change [38]. It comprises 6 primary steps: (1) assess needs and develop a logic model of the health problem; (2) develop matrices of behavioral change objectives; (3) identify theory-based methods and practical applications to design the program; (4) produce program components and materials; (5) plan for program adoption, implementation, and sustainability; and (6) plan for evaluation [38]. IM is widely used to design disease prevention and disease management interventions worldwide. A recent systematic review has demonstrated a significant increase in the uptake of disease prevention behaviors associated with IM-based interventions and placebo control groups [39]. IM has been successfully applied in the domain of sexual health, including interventions to promote increased communication between parents and young adolescents on relationships and sex [39,40]. However, few applications of IM have been reported in the context of serious games for health, and to our knowledge, none in the context of intergenerational video games for health [41,42].

## Results

### Step 1: Assess Need and Develop a Logic Model of the Problem

**Figure 1:**
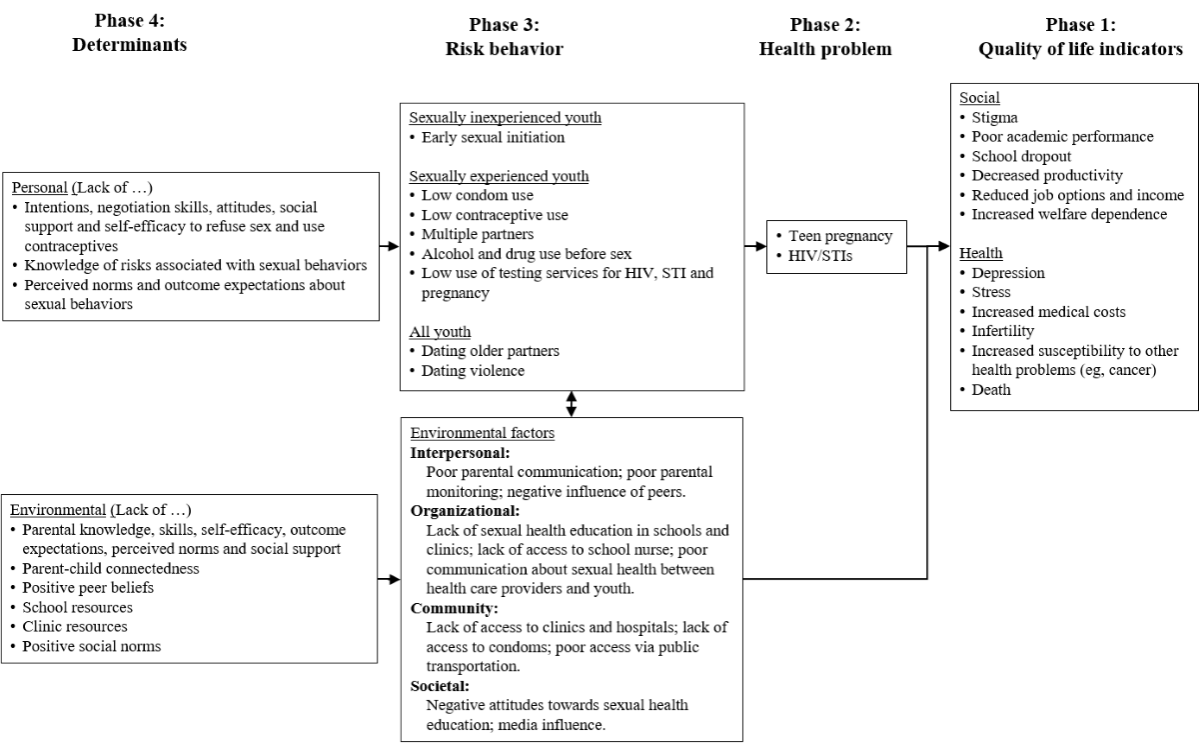
Predisposing, reinforcing, and enabling constructs in educational diagnosis and evaluation “logic model of the problem” of young adolescent sexual behavior for the Secret of Seven Stones. STI: sexually transmitted infections.

In step 1, we conducted a needs assessment to understand the health problem and priority population and to describe a theoretically- and empirically-based model for sexual health behavior (Table 1) [38]. PRECEDE (predisposing, reinforcing, and enabling constructs in educational diagnosis and evaluation) provided a framework for developing a logic model of the problem [38]. The model prescribes an *analysis of causation* for a health promotion problem that accounts for multiple ecological levels as well as the multiple determinants of a health-related behavior and environment. National statistics, data from our previous empirical studies on sexual health, qualitative data from 6 P-YAG focus groups, and a literature review of behavior change theories (principally Social Cognitive Theory, social influence models, and the theory of triadic influence) informed our development of a PRECEDE logic model describing quality of life issues and behavioral, social, and physical influences related to adolescent sexual risk behaviors (Figure 1) [28,43-49]. National statistics indicated that early sexual debut is correlated with increased risk of STIs and unintended pregnancy, increased number of sexual partners, more frequent sex, use of alcohol or drugs before sex, and reduced condom use [50-56]. Possible quality of life consequences includes increased high school dropout, welfare dependence, and negative health outcomes for children of teens [57,58]. Environmental factors include limited parent-child communication about sexual health and parental monitoring (Figure 1) [59,60].

Our needs assessment determined a priority population of young adolescents (aged 11-14 years) and their parents and program goals to increase young adolescent intentions to delay initiation sex until they are older and increase parent-child sexual health communication to delay sexual initiation. P-YAG recommendations for sexual health topics included puberty, sexual behavior, and STIs and skill building on parent-young adolescent communication, negotiation, and decision making [46]. Parents wanted to be a credible and focal resource in their child’s sexual health education, and both parents and young adolescents wanted to be more comfortable and effective in initiating and maintaining the conversation around sexual health [46]. Our needs assessment confirmed that parents and young adolescents enjoyed playing a variety of digital games, often on smartphones, and acknowledged the bonding experience of games which, when played together, were played most frequently at home [46].

### Step 2: Develop Matrices of Change Objectives

In step 2, we described the behavioral outcomes, delineated these behaviors into their component parts (performance objectives), specified behavioral determinants, and developed change (learning) objectives (Table 1).

#### Behavioral Outcomes

Drawing from our needs assessment findings and our previous studies, we identified 15 outcome behaviors that were important for the current program (Table 2) [30,31,46,61]. Outcome behaviors for young adolescents comprised delayed initiation, condom use, contraceptive use, human papillomavirus (HPV) vaccination, healthy peer and dating relationships, HIV and STI testing, parental monitoring, and young adolescent to parent communication. Decision making for sexual risk reduction can follow a self-regulation framework of s*elf-monitoring* to determine if behaviors are in accordance with one’s values and consistent with one’s goals, s*elf-judgment* to identify the presence of threats to these values, such as risky situations, s*elf-reaction* to take appropriate action (eg, avoidance or using refusal skills), and *self-evaluation* to assess the success of the chosen action [62]. Young adolescents have demonstrated capability in engaging in self-regulation that can provide a cognitive framework for navigating life challenges and reducing health risks [63-65]. Furthermore, young adolescents have demonstrated achievement in processing extensive sexual health curricula content and effectively translating this into behavioral outcomes [28,29,31]. Outcome behaviors for parents comprised parental monitoring; general parent to young adolescent communication; and parent communication about condom use, contraceptive use, HIV and STI testing, and HPV vaccination. The outcome behavior at the level of the dyad (parent and young adolescent) was dyadic communication.

**Table 2:** Outcome behaviors for young adolescents, parent, and dyad with performance objectives for the dyadic (parent-young adolescent) communication outcome behavior.

Learner–domain		Outcome behavior
**Youth^a^**		
	Healthy peer and dating relationships	Youth will have healthy peer and dating relationships
	Abstinence	Youth will not have sex
	Condom use	Youth who are sexually active or considering having sex will use condoms correctly and consistently when having sex
	Contraceptive use	Youth who are sexually active will use effective method of birth control along with condoms
	HIV and STD^b^ Testing	Youth who are sexually active will get tested and counseled for HIV and STD and unintended pregnancy
	HPV^c^ vaccination	Youth will complete the 3-dose HPV vaccination series
	Parental monitoring	Youth will establish common rules with parents about supervision and monitoring
	Youth to parent communication	Youth will communicate with their parents about dating, intimate or healthy relationships, and sexual behaviors
**Parent**		
	Parental monitoring	Parents will monitor their youth’s adherence to personal rules
	Parent to youth communication	Parents will communicate with their youth about dating, healthy intimate relationships, and sexual behaviors
	Condom use	Parents will talk to their youth about condom use when having sex
	Contraceptive use	Parents will talk to their youth about contraceptive methods
	HIV and STD testing	Parents will talk to their youth about getting tested and counseled for HIV and STD and unintended pregnancy
	HPV vaccination	Parents will talk to their youth about completing the 3-dose HPV vaccination series
**Dyad**		
	Dyadic communication	Parents and youth will interact in a mutually engaging and responsive communication process to achieve shared goals
	Performance objectives for dyadic communication	PO^d^.1: Parent and youth will pick the right time and place (T&P) to talk. PO.2: Parent and youth will converse with respect. PO.3: Parent and youth will assess the youth’s motivation to engage in the behavior under discussion. PO.4: Parent and youth will assess alternative actions to the behavior under discussion and their benefits and consequences. PO.5: Parent will share their values and expectations regarding possible behaviors (the behavior under discussion and alternate actions). PO.6: Parent and youth will develop the best plan of action together. PO.7: Parent and youth will encourage each other to keep communicating openly. PO.8: Parent and youth will reflect on what to do the same or differently next time.

^a^Youth refers to young adolescent (11-14 years).

^b^STD: sexually transmitted disease.

^c^HPV: human papillomavirus.

^d^PO: performance objective.

#### Performance Objectives

We identified 143 performance objectives (sub-behaviors) that are necessary to complete the outcome behaviors. Table 2 shows the performance objectives for dyadic communication.

##### Behavioral Determinants of Sexual Behavior

Once the target behaviors were defined, we used theory and empirical applications of theory, including our literature review and previous studies to guide the identification of determinants that likely influence successful performance. Determinants that have been described as impacting sexual behavior in young adolescents include constructs derived from the Social Cognitive Theory, social influence models, and the theory of triadic influence [28-31,47-49,61]. Programs grounded in these theories demonstrated success [28,29,31,44,45]. The determinants included behavioral capability (declarative and procedural knowledge of risk reduction and communication behaviors), skills and self-efficacy (capability and confidence to perform risk reduction and communication behaviors), outcome expectations (belief that risk reduction behaviors and communication will lead to important results), perceived norms (belief that significant others believe in and use risk reduction behaviors), and social support (recognition of social others who can assist in risk reduction; Figure 1) [59,60].

##### Behavioral Determinants of Game Play

We also attended to determinants of game play using motivational theory to optimize learner attention [66-71]. Hypothesized determinants included challenge (defined and personally meaningful goals and uncertain, difficult, and yet attainable game outcomes that are predicated on personal effort), curiosity (through novel and surprising game environments, including novel sensory stimuli, and results that can only be confirmed through play), control (through game environments and consequences that are subject to learner action), and self-efficacy (through successive positive reinforcement provided through play).

##### Matrices of Change Objectives

We defined the program learning objectives by creating separate matrices of performance objectives (row headings) and determinants (column headings) for each outcome behavior. Table 3 provides an example of a partial matrix for dyadic communication behavior (from the example in Table 2). In each matrix cell, we described the learning objective, related to a particular determinant, which contributes to achieving the performance objective. For example, in Table 3, the dyad needs to demonstrate the capability (skill determinant S1.1) to pick the right time and place to converse (performance objective). For interested readers, matrices are available from the corresponding author.

**Table 3:** Parent matrix for the dyadic parent-youth communication outcome objective that parents and children will interact in a mutually engaging and responsive communication process to achieve shared goals.

PO^a^	Determinants of behavior						
	Knowledge	Skills	Self-efficacy	Outcome expectations	Perceived norm	Perceived barriers	Social support
PO.1 Parents and child will pick the right T&P^b^ to talk	K1.1. State that the right T&P is one where both parent and child are focused and calm. K1.2. Describe the influence of emotions, preconceived thoughts, and distractions on communication. K1.3. State the importance of being aware of these influences and setting them aside before initiating communication.	S1.1. Demonstrate the ability to pick the right time and place to converse. S1.2. Demonstrate the ability to set aside emotional or cognitive predispositions before conversing.	SE1.1. Demonstrate the confidence to pick the right time and place to converse. SE1.2. Demonstrate the confidence in ability to set aside emotional or cognitive predispositions before conversing.	OE1.1. State that picking the right T&P will lead to a more focused and calm discussion. OE1.2. State that reflecting on one’s emotions, preconceived thoughts, and distractions before communication will facilitate open and respectful communication.	PN1.1 State that other parents and children have greatest communication success when they pick the right T&P.	PB1.1. State ways to overcome barriers to selecting a right T&P to communicate (schedule or environment). PB1.2. Recognize barriers to being aware of and setting aside one’s emotions or cognitions before conversing.	SS1.1. Identify others who can help in arranging a right T&P to converse.

^a^PO: performance objective.

^b^T&P: time and place.

### Step 3: Identify Theory-Based Methods and Practical Applications for Program Design

In step 3, we identified theoretical methods and practical applications to inform program design (Table 1).

#### Theory-Based Methods

A theoretical method is a general technique that influences the determinants of behaviors. If a young adolescent theoretically requires the knowledge, skills, self-efficacy, positive outcome expectations, positive perceived norms, and engagement of social support to perform sexual risk reduction and communication behaviors, then an effective sexual health education program needs to elicit positive change in these determinants. We drew from empirical literature and our previous research on sexual health and web-based curricula [38]. Table 4 provides an example of the dyadic performance objective to pick the right time and place to converse. We derived methods to increase skills and self-efficacy that comprised informing and consciousness raising, goal setting, chunking, verbal persuasion, modeling, enactive mastery, and public commitment (Table 4).

**Table 4:** Partial (example) matrix of methods and applicationsa.

Method (and theory)	Practical application	
	For youth	For parents
Information and consciousness raising	Communication activity describing PEP^b^ steps and importance of respectful communication; SDP^c^ activity teaching how to use this tool to protect personal rules; RRR^d^ to manage emotions; and “What kind of friend are you” quiz and activity.	PEP Talks 101 and SDP tip sheets with communication tips and “Ask the Expert” advice on communicating about specific topics and information on planning ahead to protect rules; and parent/youth video testimonials illustrating benefits of talking.
Goal setting (theories of self-regulation and Social Cognitive Theory)	Prompt to set personal rules before each PEP Talk and develop strategies with parent.	Personal rule to orient communication and expectation.
Chunking (information processing)	PEP to teach steps of PEP Talk; RRR to manage emotions; and SDP tool to help youth maintain personal rules.	PEP to teach steps of healthy communication and SDP tool to help youth maintain personal rules.
Verbal persuasion (Social Cognitive Theory)	SDP activity training and practice by presenting youth with pressure lines in various situations and asking youth to select appropriate response and RRR activity to manage emotions.	PEP Talk videos; parent and youth video testimonials illustrating how other parents talk to their children and describing how to talk about family values to set rules; Virtues tip sheet reviewing virtues and how to talk about them; and PEP Talk question prompts.
Modeling (Social Cognitive Theory)	Parent and youth video testimonials on communication and discussing values.	Parent and youth video testimonials on communication and discussing values.
Enactive mastery (Social Cognitive Theory)	Communication activity reviewing PEP steps and how to choose right time and place to talk; SDP activity training and practice by presenting youth with pressure lines in various situations and asking youth to select appropriate response; RRR activity to manage emotions; and prompt for youth to enter personal rules and strategies. Content progresses in terms of *maturity*.	PEP Talk videos; Virtues tip sheet reviewing virtues and how to talk about them; and PEP Talk question prompts.
Public commitment (transtheoretical model)	Discussing rules and strategies with parent during PEP Talk and then entering rules into game where they can be viewed by youth and parent throughout the game.	Rules and strategies inform social support

^a^Outcome behavior: Parents and children will interact in a mutually engaging and responsive communication process to achieve shared goals. Performance objective #1: Parents and children will pick the right time and place to talk. Determinant and change objective: Skills (S1.1) and self-efficacy (SE1.1) to pick the right time and place to converse. Youth refers to young adolescent (ages 11-14 years).

^b^PEP: partner-engage-plan.

^c^SDP: select, detect, protect.

^d^RRR: relax, rewind, replay.

Given that we were designing a serious game, we also adopted methods to influence the determinants of young adolescent persistence in game play. To address learner challenge, we designed the game to include goals to accomplish and milestones to reach and provided game scenarios of moderate difficulty [66,67,72-75]. Progress was designed to be reinforced verbally by the dojo master and with performance-based rewards (eg, higher quiz scores provide stronger battle cards and virtue tokens that can assist in winning battles) to impact self-efficacy for game play [47]. To address learner curiosity, we embedded scenarios of real-life challenges in the *fantasized* content of a gaming motif, featuring novel locations and characters [66,68,69]. We appealed to learner sensory curiosity by providing multiple modalities to convey information (sound, graphics, video, and animation), and provided opportunities for learner control with flexibility in the selection of content exposure and battles [71,76-78].

#### Practical Applications

Practical applications refer to the mode and context of program delivery that fits with the priority population. These comprise channel, scope and sequence, and theme. We designed the program to operationalize the theory-based methods and to be responsive to needs assessment recommendations from the P-YAG [46].

##### Theme

We provided 2 underlying themes. The first theme was that young adolescents do not have to “go it alone,” that the parent is a social support, dyadic communication is important, and that the discomfort and lack of confidence to discuss sexual health topics (by both young adolescent and parents) can be overcome with skills to initiate and maintain the sexual health dialogue. The second underlying theme was that young adolescents have control of their life decisions and that smart life decisions (based on self-regulation by selecting, detecting, and protecting their personal rules) have positive consequences. The game motif was a quest to liberate the citizens of the town of Seven Stones from the control of an evil villain, Frostbyte. The young adolescent is victorious if they can defeat Frostbyte in a final *boss* battle.

##### Channels

**Figure 2:**
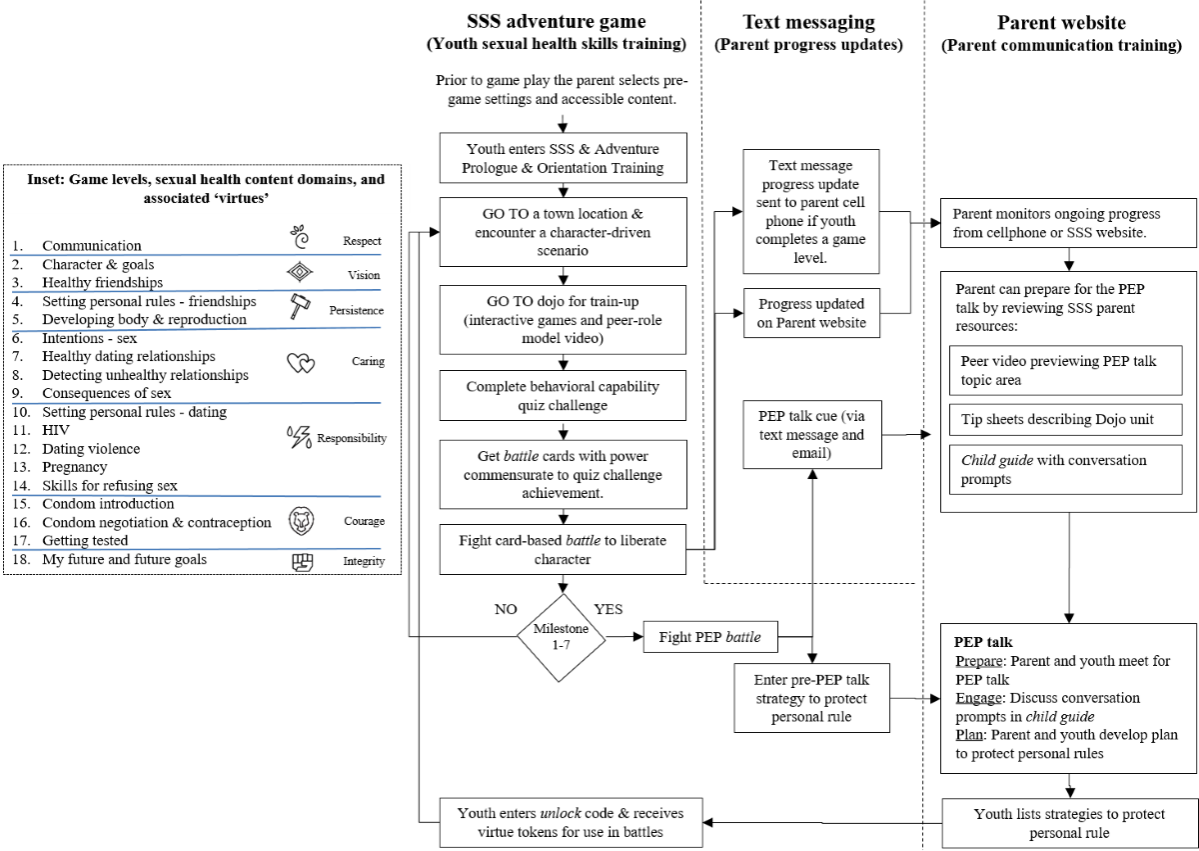
The Secret of Seven Stones program scope and sequence of play and content domains (inset). PEP: partner-engage-plan; SSS: the Secret of Seven Stones.

SSS comprised (1) an adventure game for young adolescent skills training delivered via a desktop computer, (2) a text-based notification system to provide progress updates for parents and cues to initiate dialogue with their young adolescent, and (3) a website for parent skills training and progress monitoring (Figure 2).

##### Scope and Sequence

**Figure 3:**
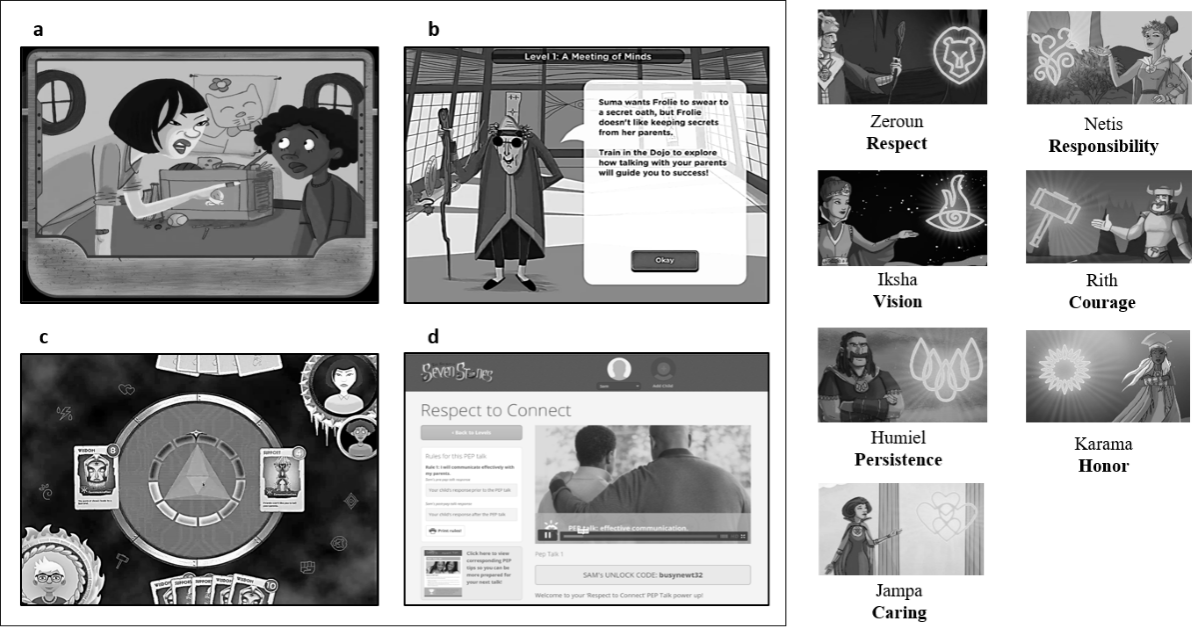
The Secret of Seven Stones (SSS) sample game and parent website screens and personified virtues. (a) Animated video introducing “Suma” and “Frollie” and the challenge scenario that requires resolution, (b) the “Dojo” (and Dojo master, Alfred) where the player receives skill training activities to prepare for “battle,” (c) card battle with wisdom, skill, and support cards to liberate Frollie, and (d) SSS parent website featuring parent resources (videos and tip sheets) to enable parent competency in partner-engage-plan talks, and an array (on right) of personification and imagery of virtues referred to in the game.

We designed the SSS interactive adventure game to provide sexual health skills training primarily for young adolescents because, although dyads supported a gaming strategy, parent time constraints would not accommodate extensive parental gameplay. As such, we designed SSS to accommodate parents in an adjunct, supporting, and gatekeeper role. The game had 18 levels to accommodate the educational content. Each game level approximated 45 to 60 min of game play and contained multiple short duration (2-5 min) activities. The SSS game sequence required young adolescents to (1) visit a location in Seven Stones and encounter citizens who are facing a sexual health challenge (eg, a conflict between being true to yourself vs maintaining a friendship); (2) enter the *Dojo* to complete educational activities, which were adapted from pre-existing evidence-based curricula and to *power-up* on relevant knowledge and skills; (3) complete a *challenge quiz* to earn battle cards of wisdom, skill, and support; and (4) initiate a card-based *battle* to liberate the citizen *opponent* (Figures 2 and 3). Dojo activities comprised interactive and noninteractive animations, mini games, role model videos, and role-playing activities from our previous curricula [28,30,31,43,61]. These activities used methods of consciousness raising, chunking, goal setting, verbal persuasion, modeling, enactive mastery, and planning coping responses and have been demonstrated to be effective in promoting behavior change in previous studies (Table 4) [28,31,43,79,80].

The program title, SSS, derives from (1) the town of Seven Stones, (2) the 7 supportive parental interactions during the quest, and (3) the 7 character virtues acquired at each of these dialogues (Figure 2 inset). SSS was designed to (1) provide a common foundation for talking points drawn from the game experience; (2) train young adolescents (and provide resources to parents) on how to communicate; (3) take a *life skills* approach that introduces character traits, healthy friendships, and rule setting long before discussing sex; and (4) providing the opportunity for ongoing dialogue to *normalize* such discussions, allowing a more subtle transition to sexual health topics over time.

The parent is updated on the young adolescents’ progress through text messages (Figure 2). At each of the 7 game *milestones* the young adolescent is *locked out* of the game and the parent is cued (via text message and e-mail) to have a PEP talk with their young adolescent. In a *PEP* talk, parents and young adolescents decide a time and location to have a face-to-face discussion (partner), discuss personal and family rules (engage), and develop goals and strategies to maintain these rules (plan). Upon completion of a PEP talk, the parent provides an *unlock* code, enabling the young adolescent to receive cards and virtue tokens to use in future battles (Figures 2 and 3).

SSS provides increased challenge by sequencing content from topics with less *mature* content early in the game (eg, healthy friendships) to more *mature* content later in the game (eg, sexual risk reduction). Early PEP talks focus on friendships and decision making to *normalize* discussions and make the transition to later discussions of reproduction and sexual relationships less abrupt and awkward. The SSS is designed to accommodate family values. A control feature allows parents to delay the delivery of more *mature* content of condoms and contraceptives to a time when they perceived their young adolescent to be developmentally ready. The SSS parent website recommends that children be exposed to all content and that content is provided sequentially ensuring foundational material is mastered before exposure to more *mature* content.

SSS was designed to respond to game preferences emanating from the focus groups. A general preference for boys was the quest to defeat Frostbyte in a boss battle, with girls to resolve interpersonal relationship conflicts among the citizens of Seven Stones, and both girls and boys to use card games for *battle*. SSS was designed to be responsive to the needs of lesbian, gay, bisexual, transgender, queer, and intersex (LGBTQI) young adolescents with activities that are were inclusive of sexual minority preference (eg, scenarios in which young adolescents can choose a partner of either gender and use gender-neutral names) and with materials (eg, fact sheets) that focus on sexual minority issues (eg, LGBTQI and sexuality defined, self-acceptance, the notion of what *normal* means, social support [“Who can I talk to?”], things to consider before coming out at home, and organization resources).

We designed an SSS website (Figures 2 and 3) to promote communication skills training for both mothers and fathers to enable parents to be a more credible resource in their young adolescents’ sexual health education. Resources comprised 15 PEP talk and communication role model videos featuring parents and young adolescents and 10 communication tip sheets. PEP talk videos were of 2-min duration, introduced the content of the PEP Talk, updated on the educational content covered by their young adolescent, and provided tips on preparing for their PEP Talk. Testimonial videos showed both mothers and fathers and young adolescent role models describing their real-world interactions and positive communication experiences. Tip sheets provided summaries of game content, strategies for engaging their young adolescents in conversation, and exercises to increase communication skills and self-efficacy.

### Step 4: Produce Program Components and Materials

In step 4, we produced and pilot tested an SSS prototype comprising the first 2 game levels (Table 1).

#### Prototype Development

We designed SSS for installation on desktop computers (both Windows and Mac) using the Adobe Interactive Runtime through a broadband connection. The back end mini Structured Query Language (MSQL) database and parent website were implemented using a web server running Hypertext Preprocessor (PHP) built on the Yii framework and accessible through standard browsers using a broadband connection. The back end database was designed to store game data allowing *pause points* that allow players to exit and re-enter SSS without loss of game progress [46].

#### Prototype Feasibility Testing

We conducted a 2-week pilot test of feasibility in the homes of 10 dyads to determine functional integrity, acceptability by parents and young adolescents, and to explore psychosocial impact. This sample size is sufficient for usability testing and comprises young adolescents (aged 11-14 years; mean 13.1, SD 1.20 years, predominantly males (7/10, 70%), and of White (5/10, 50%) and Hispanic (3/10, 30%) ethnicity [81,82]. Young adolescents were experienced with games; they reported playing for 5 to 8 hours a week (4/10, 40%), playing first-person shooter and multiplayer games (3/10, 33%), and playing on gaming consoles (6/10, 60%) and cell phones (5/10, 50%). Parents comprised mainly mothers (8/10, 80%) and reported either not having played in the last 3 months (4/10, 40%) or playing for less than 2 hours (3/10, 30%). Most played creative or casual games (10/10, 100% and 8/10, 80%, respectively) and all participants played on their cell phones (10/10, 100%).

#### Measurement

We collected parent and young adolescent self-report data using computer-assisted self-administered surveys on study laptops at baseline and at the 2-week follow-up. Our pilot study enabled the testing of protocols to be employed in subsequent efficacy testing. Parent consent and young adolescent assent were obtained before data collection. The feasibility process measures comprised system access logs and user reports (written and verbal) of program issues. Dyads rated SSS on likeability, ease of use, duration, understandability, credibility, perceived impact, and motivational appeal using previously described rating scales [30]. They also assessed the commercial potential of the SSS (discussed in step 5 below). An exploratory analysis was conducted on the impact of SSS on psychosocial determinants of parent-young adolescent communication regarding sex (perceived quality of communication, self-efficacy for parent-child communication, outcome expectations for communication, communication ability, communication openness, and parent-young adolescent connectedness) and attitudes toward using digital games for learning (using an adapted 12-item scale) [83-87]. Scales were provided as pretests before SSS use and at the 2-week follow-up. The Wilcoxon signed-rank test was used as a nonparametric alternative to a *t* test as the sample size (n=10 dyads) was too small to assume normal distribution.

#### Results of Feasibility Prototype Testing

**Figure 4:**
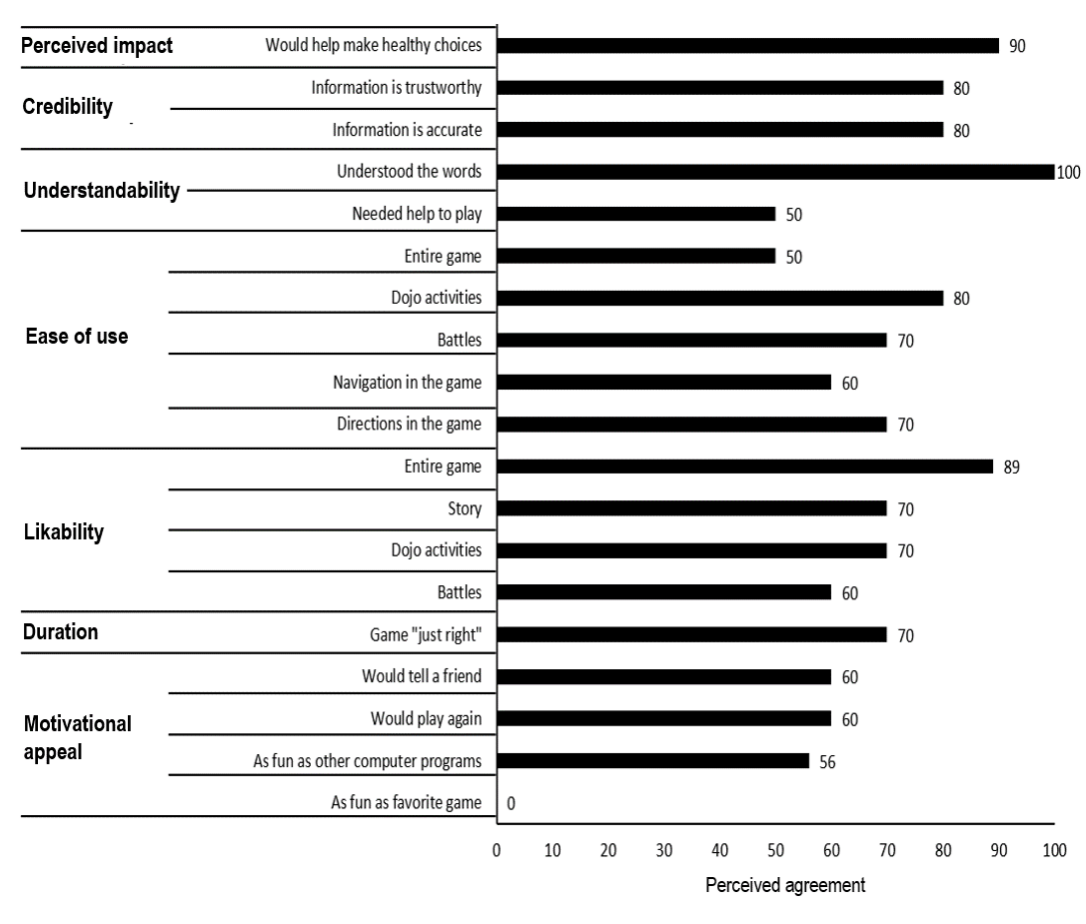
Young adolescent ratings for prototype levels 1 and 2.

The SSS prototype functioned according to specifications with players completing the 2 levels within the 2-week period. Most young adolescents rated SSS as likable and credible (6/10, 60%-10/10, 100%) and helpful in making healthy choices (9/10, 90%; Figure 4). The prototype was rated as more fun or as much fun as other sexual health lessons (5/9, 54%), but it was not rated more favorably than favorite computer games. Conversely, less young adolescents agreed that SSS was easy to use (5/10, 50%) and indicated that they needed help to play (5/10, 50%). These lower ratings were primarily associated with technical issues (reported bugs and system *crashes*), which were a source of frustration for participants and a barrier to completing the assigned activities and led to navigation difficulties where young adolescents would lose track of their next destination in the town of Seven Stones.

**Figure 5:**
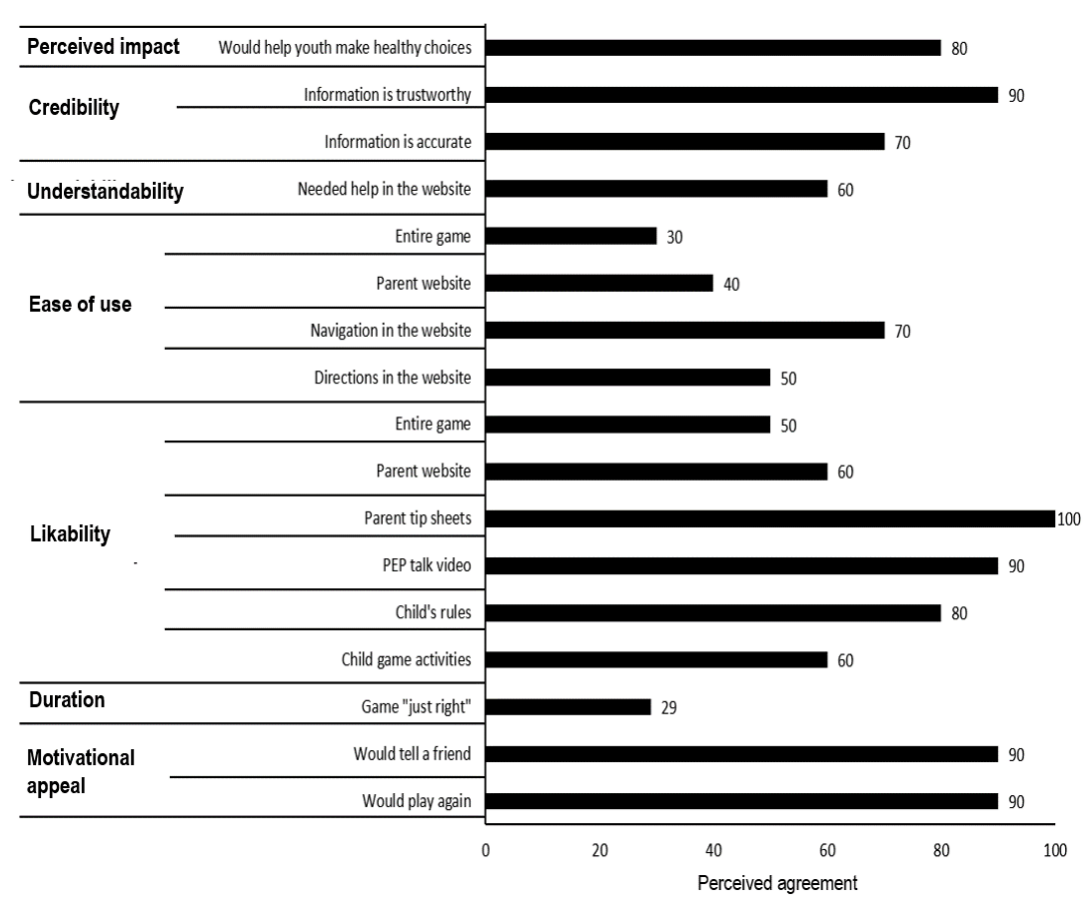
Parent ratings for parent website and prototype levels 1 and 2.

Parents rated the website as likable, easy to navigate, credible, and understandable and the game as useful in helping young adolescents make healthy choices (6/10, 60%-10/10, 100%; Figure 5). Conversely, ratings of website ease of use and acceptability were lower (4/10, 40% and 2/7, 29%, respectively) as was SSS game duration (2/7, 29%).

#### Psychosocial Variables for Communication

Exploratory analysis demonstrated positive change in young adolescent attitudes toward using computer games for learning and parent communication outcome expectations (*P*≤.05), driven principally by increased confidence and perceived ease of learning. Other psychosocial variables for communication were not significantly impacted within the 2-week test period (Table 5).

**Table 5:** Young adolescent change in dyadic communication following exposure to Secret of Seven Stones 2-level prototype

Scales	Young adolescent					Parent				
	Baseline		2-week follow-up		P value^a^	Baseline		2-week follow-up		P value^a^
	n	Mean (SD)	n	Mean (SD)		n	Mean (SD)	n	Mean (SD)	
Attitudes to computer games for learning	*10* ^b^	*1.58 (0.65)*	*10*	*1.76 (0.51)*	*.05*	N/A^c^	N/A	N/A	N/A	N/A
Communication about sex self-efficacy	7	1.35 (1.09)	9	1.67 (1.11)	.45	1^d^	2.75 (0.00)	7	2.57 (0.36)	.32
Communication about sex outcome expectation	8	2.33 (0.61)	9	2.48 (0.65)	.99	*10*	*2.31 (0.18)*	*10*	*2.47 (0.18)*	*.03*
Quality of communication about sex	8	1.91 (0.37)	7	2.01 (0.34)	.73	10	2.18 (0.25)	9	2.28 (0.29)	.07
Communication ability	10	3.80 (2.30)	9	5.22 (1.09)	.14	10	4.90 (0.57)	10	4.20 (1.40)	.15
Communication openness	8	1.43 (0.57)	8	1.41 (0.61)	.67	10	1.31 (0.21)	8	1.28 (0.22)	.72
Parent-adolescent connectedness	10	3.52 (0.57)	9	3.64 (0.52)	.85	10	3.72 (0.33)	10	3.70 (0.45)	.87

^a^Wilcoxon signed-rank.

^b^Italics indicate significance at *P*≤.05.

^c^N/A: not applicable.

^d^Respondent data missed in web-based survey.

#### SSS Completion in Preparation for Implementation

Prototype testing informed our completion of the full 18-level prototype. SSS had demonstrated feasibility and compared favorably to other sexual health education programs. However, design modifications were indicated for both the adventure game and parent website. Program *bugs* and *stalls* were tracked and fixed, and the player navigation was modified. An open world architecture allowing the player freedom to move at will and explore the gaming environment, originally supported by the P-YAG, was redesigned to be a more directed sequence of destinations with more clearly articulated instructions. Parents reported website navigational problems when trying to locate the parent information associated with their young adolescent’s game level, largely due to forgetting how to use the site between visits. To create a more intuitive site, visual cues that highlighted the relevant information were provided upon log-in. Printed and electronic parent guides were developed, and a web-based tutorial was included to improve the understanding of navigation.

### Step 5: Plan for Program Adoption, Implementation, and Sustainability

In step 5, we planned for SSS implementation and dissemination (Table 1). We developed a commercialization plan in accordance with the STTR funding mechanism, which comprises direct-to-consumer sales and bulk licensing to intermediary organizations.

#### Direct-to-Consumer Sales

Parents and young adolescents in the feasibility pilot provided information on purchase interest, cost points, barriers, and facilitators for purchase and expected marketing channels. Most young adolescents (6/10, 60%) expressed interest in purchasing SSS if it was for sale. They cited potential barriers to purchase of cost (56%), uncertainty about SSS efficacy (2/9, 22%), and long play duration (2/9, 22%). Most parents (6/10, 60%) were willing to pay at least US $20 for SSS. They cited barriers to purchase of duration (5/10, 50%) and possible misalignment with their values (4/10, 40%), potential facilitators to purchase as testimony from other parents (5/9, 56%), and evidence of effectiveness (4/9, 44%). Parents (4/10, 40%) and young adolescents (5/9, 56%) expected to hear about SSS mainly through school.

#### Bulk Licensing

Discussions with representatives from third-party distribution channels that promote family wellness (eg, WebMD, Aetna, Humana, and ActiveHealth) resulted in awareness of the SSS proof-of-concept and interest in ongoing discussion as the product matures out of prototype through efficacy testing. Additional market analysis will use the Strategyzer strategic management framework to further develop the business model [88]. Ongoing customer discovery interviews will specify marketing approaches, delivery channels, and SSS modifications to address pain points (problems to be solved), gain creators (existing solutions), and value propositions (program attributes that drive a purchase decision) across all customer segments.

### Step 6: Plan for Evaluation

In step 6, we planned to evaluate the SSS (Table 1). Our SSS evaluation plan comprised a randomized controlled efficacy trial with 85 parent-young adolescent (aged 11-14 years) dyads to test the full SSS game. Dyad psychosocial and communication data will be collected at baseline and at the third and sixth month. Hypotheses would be that dyads accessing SSS will report increased frequency and quality of communication about sexual health and young adolescents will demonstrate greater intentions to delay initiation of sexual behavior compared with those not receiving SSS.

## Discussion

### Principal Findings

The SSS represents a novel application of an intergenerational serious game for sexual health education, adding to the limited cadre of home-based programs that facilitate parent involvement in influencing young adolescent behaviors and reducing adolescent sexual risk taking [8,18,46].

SSS is an intergenerational game to the degree that it provides both parents and young adolescents’ roles in the gaming experience and encourages dialogue to accelerate game play. Parents and young adolescents could sit together to play SSS, but currently the game does not allow parents and young adolescents to synchronously play or compete in the game space. Parental time constraints necessitated a gatekeeper role for parents rather than a dual-player mode. This is consistent with what Voida et al [35] have described as the *performance/audience* role pair that was pervasive in their explorative studies of intergenerational gaming. In this instance, there is a more active performer (usually the young adolescent) and a spectator role (usually the older person). The analogy is that the SSS adventure game is experienced more actively by young adolescents and more vicariously by the parent gatekeeper.

Success in serious game design is predicated on achieving a balance between strategies for behavior change and playability. This introduces a tension between ensuring sufficient educational content and optimal exposure for behavioral impact while providing an engaging and immersive experience. As a health education program, parents positively rated SSS, reporting it to be valuable and credible. The positive impact on attitudes toward the use of computer games for learning supports the utility of this strategy. The interest of young male adolescents (7/10, 70% of our feasibility sample) to play SSS, which is a population group that has traditionally been more difficult to recruit and retain in sexual health education programs, was also encouraging [28,43]. The use of a serious gaming strategy may be intrinsically motivating to this population segment, thereby increasing exposure to sexual health content [33]. Most young adolescents reported that the duration of SSS was *just right*. This is encouraging as approximately 13 hours of sexual health curricula exposure is optimal to see the impact on delayed sexual initiation [89]. Conversely, even within the limits of a 2-level feasibility test, most parents suggested that SSS was *too long* compared with *too short* or *just right* (both 29%) despite considerable design efforts to reduce parental burden. Rectifiable *bugs* in the prototype may have contributed to this perception. It is uncertain to what degree this perception translates to reduced use or attrition of young adolescents using the full SSS program.

Young adolescents rated SSS as fun as other computer games (56% agreement) but, perhaps predictably, none rated SSS as much fun as their favorite computer game. SSS exhibited common gaming features such as the quest motif, characters and bosses, virtues, power-ups, battles, and points. However, there were dissimilarities relating to the educational content, including power-up dojo activities, quizzes, parent updates, PEP talks, and skills training around life skills issues. Furthermore, SSS could not compete with the production value of high-end commercial games that feature richly textured graphics, epic musical scores, massive scope, greater user control, and sophisticated three-dimensional game mechanics. SSS, similar to other serious games for health, may best be marketed as a palatable way to consume health information and training rather than as a direct competitor to commercially available entertainment games. Health-oriented games occupy a commercial niche that offers social value and has the potential to operate in community settings with an aligned mission (eg, schools, work places, clinics). In the context of the home, where there is an array of competing demands, the use of serious games may be more tenuous and contingent on parent and child commitment.

IM is one of a number of useful development frameworks [34,90,91]. It has been applied for game-based sexual health curricula for middle school students and interventions for gay men [28-30,38,61]. As a general development framework, it has demonstrated utility in designing, developing, implementing, evaluating, and disseminating theory-based serious games and in enabling the development of interventions that provide skills training for complex health behaviors. Commercial product development, under the NIH STTR grant mechanism, represents a novel application of the IM framework despite the burgeoning trend of innovation incubators and academic-corporate partnerships. The commercial dissemination of serious games holds the promise of generating a revenue stream to sustain them. Efforts in this arena have been ongoing for over a decade; however, research on the potential for serious games in sexual health is in its infancy and exemplary models of commercial success are yet to be reported.

### Limitations

The findings need to be interpreted in light of study limitations. The pilot study was formative in nature. A small sample size (n=10 dyads), abbreviated intervention dose (2 levels over 2 weeks), and the use of self-selected sampling, although appropriate in this setting for feasibility assessment, were insufficient to assess the efficacy of the game and impact on psychosocial and dyadic communication outcomes. The sample was inherently biased, attracting parents predisposed to improving communication with their children, and predominantly of mothers, which may have affected the parental content and resources (and hence appeal and relatability) of SSS. Our development was not powered to provide a meaningful comparison of mother and father perceptions of SSS. Mothers and fathers provided similar responses regarding the usability and feasibility of SSS (eg, that SSS was useful in helping young adolescents make healthy choices and that most parents would tell their friends about SSS). Further investigation of varied parental perspectives would be useful. It is possible that the male (father) perspective was underrepresented in the SSS development process. Postponing commencement of the field testing in favor of more extensive alpha testing may have mitigated some program *bugs* and thus some usability ratings. This is difficult to guarantee, however, and the usability data gathered was valuable despite these issues. The formative pilot testing of 2 levels, although important and useful in product evolution, inherently limited the conclusions we could offer about SSS, as it limited exposure to content topics of communication, healthy friendships, decision making, and sexual health topics that are delivered later in the game. There is a precedent in previous school-based studies to indicate that the content scope and volume is appropriate for the young adolescents, but this remains to be further empirically tested within the home context [28,29,31]. Dyads were not challenged to start having conversations on topics that traditionally cause greater discomfort, a necessary focus for the efficacy study. Program-led discussions are not always well received if they are perceived as *contrived* or *forced* and not organic. Thus, naturally occurring conversations are preferred. Future studies will focus on how parent-young adolescent dyads perceive PEP talks and whether these can promote naturally occurring discussions beyond any need for program cues. Despite limitations, the exploratory results were consistent with the content covered and indicative of the potential of exposure to the full program. The criteria by which developers pilot and field test complex interventions can vary as a function of scientific, resource, and timeline constraints, but guides exist to assist in decision making and determining evaluation designs. In this regard, a useful adjunct to IM is the United Kingdom Medical Research Council guidance document on developing and evaluating complex interventions, providing consideration of study designs with case study examples [91].

Important empirical questions remain regarding SSS. SSS allows families to choose the *match* of parent-young adolescent dyadic combinations (mother or father with daughter or son) based on existing communication dynamics, desire, and logistics. Young adolescents may prefer same-sex caregiver support for sexual health discussions. Further studies pertinent to the field of intergenerational gaming could investigate the differential benefits of alternate dyadic combinations, triadic combinations, and inclusion of siblings in improving communication and family dynamics. Altering the game mechanics to allow for text messaging updates and prompts for 2 parents and adjusting the expected communication dynamics to be inclusive of *parents* and young adolescents are relatively easy adjustments. Future studies could also contribute to our understanding of the *optimal* exposure to achieve behavioral impact.

### Conclusions

SSS provides a feasible strategy to overcome parent and young adolescents discomfort about discussing sexual health topics and enhancing the skills required to initiate and maintain this dialogue. IM is a useful framework for developing a theoretically and empirically based intergenerational, sexual health computer game (SSS) for in-home use. Further testing to assess the efficacy of the complete SSS program on parent-young adolescent communication is indicated.

## Abbreviations

HPV: human papillomavirus
IM: intervention mapping
LGBTQI: lesbian, gay, bisexual, transgender, queer, and intersex
NIH: National Institutes of Health
PEP: partner-engage-plan
PRECEDE: predisposing, reinforcing, and enabling constructs in educational diagnosis and evaluation
P-YAG: Parent-Youth Advisory Group
SSS: Secret of Seven Stones
STI: sexually transmitted infection
STTR: Small Business Technology Transfer Research
